# Comparing the Accuracy of Ohmann and Alvarado Scoring Systems in Detection of Acute Appendicitis; a Cross-Sectional Study

**DOI:** 10.22037/aaem.v9i1.1187

**Published:** 2021-05-05

**Authors:** Seyed Jalal Eshagh Hoseini, Mostafa Vahedian, Alireza Sharifi

**Affiliations:** 1 Department of Surgery, School of Medicine, Shahid Beheshti Hospital, Qom University of Medical Sciences, Qom, Iran.; 2 Research Center for Environmental Pollutants, Qom University of Medical Sciences, Qom, Iran.; 3 Student research committee, Qom University of Medical Sciences, Qom, Iran.

**Keywords:** Appendicitis, Decision Support Systems, Clinical, Data Accuracy, Comparative Study

## Abstract

**Introduction:**

Alvarado Scoring System (ASS) and Ohmann Scoring System (OSS) are two scoring systems for diagnosing acute appendicitis (AA). This study was designed to compare the diagnostic accuracy of the two mentioned scores in detection of acute abdominal cases at risk for AA.

**Methods:**

In this prospective cross-sectional study, patients admitted to the emergency departments of two academic hospitals in Qom, Iran, with right lower quadrant (RLQ) abdominal pain suspected to AA were evaluated. All cases were scored using ASS and OSS, and screening performance characteristics of the two scores were calculated and reported considering the pathologic findings as a gold standard.

**Results:**

174 patients with a preliminary AA diagnosis and the mean age of 38.1 ± 10.63 years (11- 73) were included in this study (62.07% male). At the optimal cutoff point of ≥7 for the ASS, the sensitivity and the specificity were 46.43% (95% CI: 37.97%-55.07%), 97.05% (95% CI: 84.67%-92.93%), respectively. At the optimal cutoff point of ≥11 for the OSS, the sensitivity and the specificity were 74.29% (95% CI: 66.22%-81.29%), and 55.88% (95% CI: 37.89% - 72.82%), respectively.

**Conclusion:**

Based on the finding of this study, the ASS ≥ 7 was more accurate than the OSS ≥ 11 for detection of AA. But it should be considered that the overall accuracy of Alvarado and Ohmann scores in this regard were fair (0.83) and poor (0.67), respectively.

## 1. Introduction:

The most common cause of acute abdominal pain is Acute Appendicitis (AA) with a lifetime risk of 7–8% (1, 2). It has dangerous complications such as perforation and peritonitis, which can be associated with morbidity and mortality (1). The incidence of AA is stable in most Western countries, but recent studies show that AA is rapidly rising in newly industrialized countries (3). Ghasemian M. et al. study, performed in 2019, reported that the annual incidence of AA in Birjand (Iran) was 12.74 per 10000 population (4). AA most often happens in individuals with the age of 10-30 years and it occurs in males and females with a ratio of approximately 1.4:1(5). Although AA has a high incidence, its diagnosis can often be a challenge for physicians as the classic presentations of AA, such as periumbilical pain followed by vomiting, nausea and Rovsing’s sign, happen in only 50-60% of cases (6).

There is no single symptom, sign, or diagnostic tool to accurately confirm the diagnosis (7); therefore, the diagnosis of AA is based on a combination of history, physical examination, and laboratory findings, supplemented by selective focus imaging (8). Imaging modalities such as radiography, ultrasound, CT scan, and MRI are used to diagnose AA (9, 10) but there is major controversy over their diagnostic role (8). In an attempt to improve the accuracy of AA diagnosis, several scoring systems such as Alvarado Scoring System (ASS) and Ohmann Scoring System (OSS) have been developed to systematically combine laboratory values, physical exam findings, symptoms and patient characteristics (6).

ASS was first introduced in 1986. It is the most reported scoring system for evaluating the AA (6). Another common scoring system is OSS that includes seven clinical parameters and a White Blood Cell (WBC) count (11). Different studies report different diagnostic accuracies for these scoring systems, for example, Yilmaz et al. study in 2017 (12) reported that ASS and OSS can be useful for predicting AA, but Arzu Sencan et al. study in 2014 (13) stated that their specificity and sensitivity were not sufficient for diagnosing AA. Although ASS and OSS are inexpensive, easy to use and reproducible with high success rates, they still have not become the main part of routine practice (14). So, this study was designed to compare the diagnostic accuracy of Alvarado and Ohmann scores in detection of acute abdominal cases in favor of AA.

## 2. Methods:

***2.1. Study design and setting***

In this prospective cross-sectional study, patients who were admitted to the emergency department of Nekuee and Shahid Beheshti Hospitals, Qom, Iran, with right lower quadrant (RLQ) abdominal pain suspected to AA, during one year, were evaluated. This study was approved by the ethics committee of Qom University of Medical Sciences (Ethics code: D/24/350). All subjects consented to participate in the study, and the data were recorded by emergency department (ED) physicians.

***2.2. Participants***

All patients above 11 years old with RLQ abdominal pain suspected to AA and candidate for appendectomy, who were referred to ED during the study period, were included using non-probability sampling method. The exclusion criteria were as follows: (a) being under 11 years of age, (b) elective appendectomy, (c) pregnancy, (d) phlegmon formation, (e) existence of abdominopelvic malignancy.

***2.3. Data gathering***

The following data were recorded for all subjects: Complaints at the time of admission, physical examination, and laboratory findings. The obtained data were used to calculate Alvarado and Ohmann scores by a surgeon before appendectomy. The parameters of ASS and OSS are demonstrated in appendix 1. All patients underwent appendectomy and were categorized into two groups according to histopathologic diagnosis: positive appendectomy (PA) and negative appendectomy (NA).

ASS evaluates 8 parameters, which include symptoms, clinical findings, and leukocyte count. The highest possible score is 10, and our optimal cutoff point for appendectomy was ≥ 7.

 OSS is also composed of 8 parameters (Tenderness in right lower quadrant, rebound tenderness, presence of urinary system complaint, character of pain, re-localization of pain to the right lower quadrant, age, leukocyte count, and abdominal rigidity). The highest possible score is 16, and we recommended appendectomy for scores ≥ 11.

***2.4. Statistical Analysis***

The data were analyzed using SPSS version 22.0. Descriptive statistics for categorical data were expressed as numbers and percentages and median (minimum-maximum) were used to express continuous data based on normal distribution. Student’s t-test was used for variables with normal distribution. The screening performance characteristics of the scoring systems were measured. A greater area under the receiver operating characteristic (ROC) curve (AUC) indicates better diagnostic value. p<0.05 was considered statistically significant. Sensitivity, specificity, positive predictive value (PPV), negative predictive value (NPV), positive likelihood ration (PLR) and negative likelihood ration (NLR), and accuracy with 95% confidence interval were calculated and compare between two scoring systems. In the present study, area under the curve of 90 to 100 was considered as excellent accuracy, 80 to 90 as good, 70 to 80 as fair, 60 to 70 as poor, and 50 to 60 as fail.

## 3. Results:

***3.1. Baseline characteristics of studied cases***

Appendectomy was performed for 181 patients with suspicion to AA but after histopathology consideration, 7 patients were excluded because they had tumoral appendicitis. Therefore, 174 patients with a preliminary AA diagnosis and the mean age of 38.1 ± 10.63 years (11- 73) were included in this study (62.07% male). Table 1 shows the baseline characteristics of studied cases. The diagnosis of AA was histopathologically confirmed in 136 cases (78.16%) and 38 (21.84%) patients had negative appendectomy. 

***3.2. Comparing the scores***

**Figure 1:**
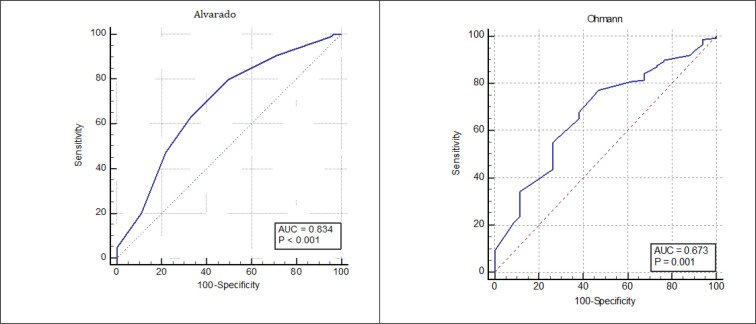
The area under the receiver operating characteristic (ROC) curves of Alvarado and Ohmann scoring systems (p = 0.001).

The median ASS and OSS scores were 7 (2 - 10) and 14 (3 - 16), respectively. Screening performance characteristics of the studied systems in determining the cases with AA are presented in table 2 and figure 1. Sensitivity and specificity of OSS in the cut-off value of ≥11 were 74.29% (95% CI: 66.22 - 81.29), and 55.88% (95% CI: 37.89 - 72.82), respectively. For ASS, these measures were 46.43% (95% CI: 37.97 - 55.07) and 97.05% (95% CI: 84.67 - 92.93), respectively. Area under the ROC curves of ASS and OSS in detection of AA cases were 0.83 (95% CI: 0.77- 0.88) and 0.67 (95% CI: 0.598 to 0.742), respectively (p = 0.001).

## 4. Discussion

In our study, OSS’s (cut off point ≥11) sensitivity, specificity, and PPV and NPV were 74.29%, 55.88%, 87.4%, and 34.5%, respectively. Also, ASS’s (cut off point ≥7) sensitivity, specificity, and PPV and NPV were 46.43%, 97.6%, 98.5%, and 30.6%, respectively. Based on the results of the current study, OSS has higher sensitivity and NPV than Alvarado scoring system in diagnosis of AA, but Alvarado score has higher specificity and PPV than Ohmann score. 

AA is the most common reason for abdominal pain in cases seen in surgery clinics and emergency services but its definitive diagnosis can be difficult. Delayed diagnosis and intervention in patients with AA can lead to high-risk complications such as perforation, sepsis, and morbidity and mortality (12). Appendectomy is performed in 12 to 23% of all people during their lifetime (15). On the other hand, the rate of unnecessary negative appendectomy based on the intraoperative and histopathological results is high (about 12% -40%). However, there are now many diagnostic scoring systems such as ASS and OSS, as well as advanced imaging methods to decrease the rate of negative appendectomies (12). 

In Chong CF et al. study, ASS has been reported to be less specific in Asian populations compared to American/European populations (7). The present study was designed to compare the ability of ASS and OSS in predicting AA. 

ASS is thought to have high sensitivity and specificity (12). Many studies have been done affirming validity for predicting AA. For example, In Schneider et al. study (16), Alvarado score ≥7 had sensitivity and specificity of 72% and 82%, respectively. In another study conducted by Hamid Kariman et al. (17), Alvarado score greater than 7 yielded 37% sensitivity and 95.65% specificity. The sensitivity and specificity of ASS in our study were higher than those reported in Kariman and Schneider studies. 

OSS is an easy and useful system employed in diagnosis of AA (18). Hassan Erdem et al. study (19) reported sensitivity, specificity, PPV and NPV of OSS (cutoff point = 12) as 96%, 42%, 78% and 83%, respectively. In another study by Sanjay N Koppad et al. (11) the sensitivity, specificity, PPV, and NPV of OSS (cutoff point = 12) were 96%, 66.7%, 82.8, and 90.9%, respectively. The reported PPV in our study was higher than these studies. 

Some studies have compared ASS and OSS in diagnosis of AA. In Korkut et al. study in 2020 (14), 74 Patients were categorized into two groups according to their histopathological results: positive and negative appendectomy. The accuracy of different scoring systems in diagnosis of the AA was investigated. Sensitivity, specificity, PPV and NPV were 71.9%, 89.9%, 97.92%, 30.77%, respectively, for OSS; versus 60.9%, 89.9%, 97.56% and 24.24%, respectively, for ASS. Their study reported a higher accuracy rate for OSS. In another study done by Yilmaz et al.(12), a total of 105 patients diagnosed with AA were enrolled, then OSS and ASS scores were calculated, separately. They concluded that ASS can predict AA better than OSS, while OSS was more useful for providing guidance in diagnosing AA when conditions are more obscured and uncertain.

In a review study conducted by Daniel LH et al. in 2017, the classical picture of shifting pain associated with nausea, vomiting, and anorexia happens in less than half of presentations (20), while in our study, shifting pain, vomiting, and loss of appetite percentile is reported in 73.61% (n=106), 59.19% (n=103) and 70.68% (n=123) of cases, respectively. Abdominal pain has been introduced as the most common manifestation (21), which is in line within our study result (89.65% (n=156)). Based on our study results, the most common symptoms and signs were RLQ pain (n=156), RLQ tenderness (n=151), and loss of appetite (n=123), respectively. There is not any specific bloods test for AA. However, if there is an increased WBC, C reactive protein (CRP), proportion of polymorphonuclear (PMN) cells, or granulocyte count, then AA is more likely (21); while leukocytosis is reported in 71.26%(n=124) of the cases in our study.

Considering the fair and poor accuracy of studied scoring systems, it seems that ASS and OSS need further modifications to be more accurate than what they are now to be applicable in routine practice. 

**Appendix 1:** Parameters of Alvarado and Ohmann scoring systems

**Alvarado parameters**	**Score**	**Ohmann parameters**	**Score**
Migration RLQ pain	1	Tenderness in RLQ	4.5
Anorexia	1	Rebound tenderness	2.5
Nausea and vomiting	1	Absence of urinary complications	2
RLQ tenderness	2	Steady pain	2
Rebound tenderness	1	White blood cell count >10000/cm^3^	1.5
Fever (>37.3)	1	Age <50 years	1.5
Leukocytosis	2	Migration RLQ pain	1
Shift to the left of neutrophils	1	Rigidity	1
**Total**	**10**	**Total**	**16**

RLQ: right lower quadrant.

**Table1:** Baseline characteristics of studied patients

**Variables **	**Values (n= 174)**
**Age (year)**	
Mean ± SD	38.1 ± 10.63
**Gender **	
Male	108 (62.07)
Female	66 (37.93)
**Appendectomy findings **	
Positive	136 (78.16)
Negative	38 (21.84)
**Scoring systems parameters **	
RLQ tenderness	156 (89.66)
Migrating RLQ pain	10 (60.92)
Anorexia	12 (70.69)
Nausea and vomiting	103 (59.2)
Shift to the left of neutrophils	85 (48.85)
Rebound tenderness	11 (64.37)
Rigidity	54 (31.03)
Fever (>37.3)	62 (35.63)
Leukocytosis	124 (71.26)
Negative urine complications	142 (81.61)
Steady pain	120 (68.97)
Age< 50 year	151 (86.78)

Data are presented as mean ± standard deviation (SD) or frequency (%). RLQ: right lower quadrant.

**Table2:** Screening performance characteristics of Alvarado and Ohmann scoring systems in prediction of acute appendicitis in emergency department

**Character**	**Alvarado (** **≥** ** 7)**	**Ohmann (** **≥** ** 11)**
True positive	65	104
False positive	1	15
True negative	33	19
False negative	75	36
Sensitivity	46.43 (37.97 - 55.07)	74.29 (66.22 - 81.29)
Specificity	97.05 (84.67 - 92.93)	55.88 (37.89 - 72.82)
Positive predictive value	98.5 (90.34 - 99.78)	87.4 (82.43 - 91.11)
Negative predictive value	30.6 (27.17 - 34.16)	34.5 (25.93-44.31)
Positive likelihood ratio	15.77 (2.27-109.76)	1.68 (1.14 - 2.50)
Negetive likelihood ratio	0.55 (0.47 - 0.65)	0.46 (0.31 - 0.69)

Data are presented with 95% confidence interval.

## 5. Limitations

The small sample size and intrinsic shortcomings of cross-sectional studies were among the most important limitations of the present study.

## 6. Conclusion

Based on the finding of this study, ASS ≥ 7 was more accurate than the OSS ≥ 11 for detection of AA. But it should be considered that the overall accuracy of Alvarado and Ohmann scores in detection of AA were fair and poor, respectively. 

## 7. Declarations

### 7.1. Author contribution

Study concept and design and analysis and interpretation of data: S.J.E.H.; drafting of the manuscript: A.SH.; statistical analysis and interpretation of data M.V.; critical revision of the manuscript for important intellectual content: A.SH.

### 7.2. Ethical approval

Ethics committee approval was received for this study from Qom University of Medical Sciences.

### 7.3. Funding/Support

We did not receive any funding and support for this study.

### 7.4. Conflict of interest

There is no conflict of interest for authors.
